# Supplementary material for: Opposing roles for DNA replication initiator proteins ORC1 and CDC6 in control of Cyclin E gene transcription
Source: eLife. 2016 Jul 26;5:e12785. doi: 10.7554/eLife.12785 (PMC4987141; doi:10.7554/eLife.12785)
Supplement: Supplementary file 1. — DOI: http://dx.doi.org/10.7554/eLife.12785.019 [file elife-12785-supp1.docx]

**Supplementary Table 1. Oligonucleotides employed for this research.**

| **Primer sequences used for RT-PCR** | | | |
| --- | --- | --- | --- |
| ORC1 | | | FOR 5’-ACTGAGGCTGCATGTTTCTG-3’  REV 5’- CTCCGGTATGGTCAAGGAGT-3’ |
| CCNE1 | | | FOR 5’-ACAGATTGCAGAGCTGTTGG -3’  REV 5’-AAATGATACAAGGCCGAAGC-3’ |
| CCNA2 | | | FOR 5’-ACAAAGCTGGCCTGAATCAT -3’  REV 5’- GTGGTGCTTTGAGGTAGGTCT -3’ |
| GAPDH | | | FOR 5’- CCTGACCTGCCGTCTAGAAA-3’  REV 5’-CTCCGACGCCTGCTTCAC-3’ |
| ACTIN | | | FOR 5’-TTCAACACCCCAGCCATGT-3’  REV 5’-GCCAGTGGTACGGCCAGA-3’ |
| **Primer sequences used for Cloning (restriction sites in bold)** | | | |
| GFP-RB | FOR 5’-GAGGAG**GGATCC**ATGCCGCCCAAAACCCCCCGA-3’  REV 5’-GAGGAG**GTCGAC**TCATTTCTCTTCCTTGTTTGAGGT-3’ | | |
| MBP-RB | FOR 5’-GAGGAG**GGATCC**ATGCCGCCCAAAACCCCCCGA-3’  REV 5’-GAGGAG**GTCGAC**TCATTTCTCTTCCTTGTTTGAGGT-3’ | | |
| GST-RB | FOR 5’-GAGGAG**GGATCC**ATGCCGCCCAAAACCCCCCGA-3’  REV 5’-GAGGAG**GTCGAC**TCATTTCTCTTCCTTGTTTGAGGT-3’ | | |
| ORC1-Flag | FOR 5’-GAGGAG**GGATCC**ATGGCACACTACCCCACAAGGCTG-3’  REV 5’-GAGGAG**GTCGAC**TTACTTATCGTCATCGTCCTTGTAG  TCGCCGCCGCCCTCGTCTTTCAGCGCATA-3’ | | |
| MBP-ORC1 | FOR 5’-GAGGAG**GGATCC**ATGGCACACTACCCCACAAGGCTG-3’  REV 5’-GAGGAG**GTCGAC**TTACTCGTCTTTCAGCGCATA-3’ | | |
| GFP-ORC1 | FOR 5’-GAGGAG**GGATCC**ATGGCACACTACCCCACAAGGCTG-3’  REV 5’- GAGGAG**GTCGAC**TTACTCGTCTTTCAGCGCATA -3’ | | |
| MBP-GFP-ORC1 | FOR 5’-GAGGAG**GGATCC**GAAATGGTGAGCAAGGGCGAGGAG-3’  REV 5’- GAGGAG**GTCGAC**TTACTCGTCTTTCAGCGCATA -3’ | | |
| ORC1-Flag (1-700aa) | FOR 5’-GAGGAG**GGATCC**ATGGCACACTACCCCACAAGGCTG-3’  REV 5’-GAGGAG**GTCGAC**TCACTTATCGTCATCGTCCTTGTAGTCGCC  GCCGCCGGCCTTTAGATGCTTGAGCCG -3’ | | |
| ORC1-Flag (1-768aa) | FOR 5’-GAGGAG**GGATCC**ATGGCACACTACCCCACAAGGCTG-3’  REV 5’-GAGGAG**GTCGAC**TCACTTATCGTCATCGTCCTTGTAGTCGCC  GCCGCCGATGGCCGTGATGTATGATGATGAAAACAT-3’ | | |
| GFP-ORC1 (1-700aa) | FOR 5’-GAGGAG**GGATCC**ATGGCACACTACCCCACAAGGCTG-3’  REV 5’-GAGGAG**GTCGAC**TCAGGCCTTTAGATGCTTGAGCCG-3’ | | |
| GFP-ORC1 (1-768aa) | FOR 5’-GAGGAG**GGATCC**ATGGCACACTACCCCACAAGGCTG-3’  REV 5’-GAGGAG**GTCGAC**GATGGCCGTGATGTATGATGATG  AAAACAT-3’ | | |
| ORC3-Flag | FOR 5’-GAGGAG**GGATCC**ATGGCTACGTCCTCGATGTCT-3’  REV 5’-GAGGAG**CTCGAG**TCACTTATCGTCATCGTCCTTGTAGTCG  CCGCCGCCGCAGCCTCCCCATGTTAGTCTT-3’ | | |
| ORC4-Flag | FOR 5’-GAGGAG**GGATCC**GGCATGGGCAGCAGTCGTAAATCAAAG-3’  REV 5’-GAGGAG**GTCGAC**TCACTTATCGTCATCGTCCTTGTAGT  CGCCGCCGCCTAACCAGCTTAGTGAGGA-3’ | | |
| GST-HDAC1 | FOR 5’-GAGGAG**GGATCC**ATGGCGCAGACGCAGGGCACC-3’  REV 5’- GAGGAG**GTCGAC**TCAGGCCAACTTGACCTCCTCCTT-3’ | | |
| GST-HP1α | FOR 5’-GAGGAG**GGATCC**GGCATGGGAAAGAAAACCAAGCGG  ACA-3’  REV 5’-GAGGAG**GTCGAC**TTAGCTCTTTGCTGTTTCTTTCTC-3’ | | |
| GST-CDC6 | FOR 5’-GAGGAG**GGATCC**ATGCCTCAAACCCGATCCCAG-3’  REV 5’-GAGGAG**CTCGAG**TTAAGGCAATCCAGTAGCTAAGAT-3’ | | |
| GFP-CDC6 | FOR 5’-GAGGAG**GGATCC**ATGCCTCAAACCCGATCCCAG-3’  REV 5’-GAGGAG**CTCGAG**TTAAGGCAATCCAGTAGCTAAGAT-3’ | | |
| GST-SUV39H1 | FOR 5’-GAGGAG**GGATCC**ATGGCGGAAAATTTAAAAGGC-3’  REV 5’-GAGGAG**CTCGAG**TCAGAAGAGGTATTTGCGGCAGGA-3’ | | |
| T7-SUV39H1 | FOR 5’-GAGGAG**GGATCC**ATGGCTAGCATGACTGGTGGACAGC  AAATGGGTGGGGGGATGGCGGAAAATTTAAAAGGC-3’  REV 5’-GAGGAG**CTCGAG**TCAGAAGAGGTATTTGCGGCAGGA-3’ | | |
| GFP-SUV39H1 | FOR 5’-GAGGAG**GGATCC**ATGGCGGAAAATTTAAAAGGC-3’  REV 5’-GAGGAG**CTCGAG**TCAGAAGAGGTATTTGCGGCAGGA -3’ | | |
| GST-SUV39H1 (1-240aa) | FOR 5’-GAGGAG**GGATCC**ATGGCGGAAAATTTAAAAGGC-3’  REV 5’-GAGGAG**CTCGAG**TCAACCCTTCTGTACCACACGATT-3’ | | |
| GST-SUV39H1 (132-412aa) | FOR 5’-GAGGAG**GGATCC**GAGCAGGAGCTCAATGCCAAG-3’  REV 5’-GAGGAG**CTCGAG**TCAGAAGAGGTATTTGCGGCAGGA-3’ | | |
| GST-SUV39H1 (220-412aa) | FOR 5’-GAGGAG**GGATCC**TACGAGTGCAACTCCCGCTGC-3’  REV 5’-GAGGAG**CTCGAG**TCAGAAGAGGTATTTGCGGCAGGA-3’ | | |
| **Primer sequences used for Mutagenesis** | | | |
| RB R661W | | FOR 5’-TATCGGCTAGCCTATCTCTGGCTAAATACACTTTGTGAA-3’  REV 5’-TTCACAAAGTGTATTTAGCCAGAGATAGGCTAGCCGATA-3’ | |
| RB N757F | | FOR 5’-TCTATTATAGTATTCTATTTCTCGGTCTTCATGCAGAGA-3’  REV 5’-TCTCTGCATGAAGACCGAGAAATAGAATACTATAATAGA-3’ | |
| ORC1 LxCxE | | FOR 5’-GCTGTACCTGAGTCTCTTCCCGGTCGGAAACAGGAATTC  CAAGACATCTAC-3’  REV 5’-GTAGATGTCTTGGAATTCCTGTTTCCGACCGGGAAGAGAC  TCAGGTACAGC-3’ | |
| SUV39H1 Mut (H324K) | | FOR 5’-ATCTCCCACTTTGTCAACAAAAGTTGTGACCCCAACCTG-3’  REV 5’-CAGGTTGGGGTCACAACTTTTGTTGACAAAGTGGGAGAT-3’ | |
| CDC6 A-A | | FOR 5’-CATACACTTAAGGGAGCAGCATTGGTATTTGACAATC-3’  REV 5’-GATTGTCAAATACCAATGCTGCTCCCTTAAGTGTATG-3’ | |
| **Primer sequences used for ChIP** | | | |
| -1462 to -1318 | | FOR 5’-GAGTACAGAGGGTACTCT- 3’  REV 5’-ATAGTGCCTGGACCAAGG- 3’ | |
| -683 to -575 | | FOR 5’-ATGAATGAACGACCCAATGC- 3’  REV 5’-GGCCTAGAACCAAGGCTTCA- 3’ | |
| -456 to -357 | | FOR 5’-GCGGTGCTCCTCGGGTAG- 3’  REV 5’-CTGGCGCGGGTGGAATGT- 3’ | |
| -342 to -195 | | FOR 5’-GCCGCTCCAGCGCCGCTC- 3’  REV 5’-CAGGGCCTGCGCGCCGAG- 3’ | |
| -280 to -143 | | FOR 5’-CCAGACTTCTCCCGCGTC- 3’  REV 5’-GCAGGGACGGGGAATCAG- 3’ | |
| -159 to +63 | | FOR 5’-TGATTCCCCGTCCCTGCGCCT- 3’  REV 5’-CGCTGGCGGCCGCGCCGGCTG- 3’ | |
| +350 to +490 | | FOR 5’-GTGGAGGGGACACTGAGG- 3’  REV 5’-CAGGAAGATGGCCGATGG- 3’ | |
| +816 to +944 | | FOR 5’-GTGACCGTTGTGAGTACA- 3’  REV 5’-GCTGATCAGGTTATGAGC- 3’ | |
| **siRNA Sequences** | | | |
| ORC1 siRNA #1 | | | 5’-CUGCACUACCAAACCUAUATT- 3’ |
| ORC1 siRNA #2 | | | 5’-AAGUGGAGGCGGUAGGAAATT- 3’ |
| SUV39H1 siRNA #1 | | | 5’- ACCUCUUUGACCUGGACUATT-3’ |
| SUV39H1 siRNA #2 | | | 5’- GGUGAAAUGGCGUGGAUAUTT-3’ |
| CDC6 siRNA #1 | | | 5’- CUCCAGUGAUGCCAAACUA-3’ |
| CDC6 siRNA #2 | | | 5’- CUUCCCACCUUAUACCAGA- 3’ |
| GFP siRNA | | | 5’-GCAAGCUGACCCUGAAGUUC-3’ |
