# Supplementary material for: Opposing roles for DNA replication initiator proteins ORC1 and CDC6 in control of Cyclin E gene transcription
Source: eLife. 2016 Jul 26;5:e12785. doi: 10.7554/eLife.12785 (PMC4987141; doi:10.7554/eLife.12785)
Supplement: Supplementary file 2. — DOI: http://dx.doi.org/10.7554/eLife.12785.020 [file elife-12785-supp2.docx]

**­­­­­­­­­­­­­­Supplementary Table 2. DNA polymerases used for each primer pair in ChIP analysis.**

**____________________________________________________________**

**Primer Nucleotide position DNA Polymerase**

A -1462 to -1318 LightCycler 480

B -683 to -575 Herculase Enhanced

C -456 to -357 Herculase Enhanced

D -342 to -195 LightCycler 480

E -280 to -143 Herculase Enhanced

F -159 to +63 **Herculase II Fusion**

G +350 to +490 Herculase Enhanced
H +816 to +944 Herculase Enhanced

_______________________________________________________________

The primer pairs used for chromatin immunoprecipitation and PCR analysis are shown and correspond to the positions in the *CCNE1* promoter shown in Figure 3A. The reaction conditions and DNA polymerases used to amplify this very GC rich promoter were optimized for each primer pair and the polymerases were: Herculase Enhanced DNA Polymerase (Cat No. 600262, Agilent Technologies); **Herculase II Fusion DNA Polymerase** (Cat No. 600679,Agilent Technologies) and LightCycler 480 SYBR Green I Master (04707516001, Roche).
